# Do we need prequalification of AI as a medical device to drive equitable adoption

**DOI:** 10.1038/s41746-025-02151-7

**Published:** 2025-12-17

**Authors:** Bilal A. Mateen, Bernard Aryeetey, Delese Mimi Darko

**Affiliations:** 1 PATH, Seattle, Washington, USA; 2 University of Birmingham, Birmingham, UK; 3 Food & Drug Administration, Accra, Ghana; 4 African Medicines Agency, Kigali, Rwanda

**Keywords:** Business and industry, Health care, Mathematics and computing, Medical research, Scientific community

## Abstract

Artificial intelligence offers transformative potential for global health, yet regulatory disparities threaten equitable adoption. This brief examines whether the World Health Organization’s Prequalification (PQ) framework should extend to AI. We highlight limitations of applying static product paradigms to dynamic systems, outline targeted reforms, and propose a reimagined PQ4AI that balances global technical assurance with local regulatory accountability to foster safe, effective, and equitable deployments.

## Introduction

As the US FDA, UK MHRA, European Medicines Agency (EMA), and others have attempted to rapidly adapt to accommodate the proliferating number of software-based medical devices that incorporate AI (hereafter referred to as AI-as-a-medical-device/AIaMD), less well-resourced regulators have noticeably lagged behind^1^. In some cases, this lack of regulatory clarity (especially in low- and middle-income countries (LMICs), which often have the most to gain from AIaMD due to their resource constraints) serves as a disincentive for product developers to bring solutions to market locally. In others, it means prolific deployment of AIaMD without adequate quality assurance or ongoing oversight. Historically, the World Health Organisation’s (WHO) prequalification (PQ) programme has played an essential role in addressing these issues and providing a clear path to market for healthcare innovations (in less well-resourced settings)^2^. Motivated by the WHO’s first foray into prequalifying AIaMD (See Box 1), this policy brief examines whether expanding prequalification to AIaMD is the most effective way to ensure equitable access to this novel technology.

Box 1 WHO’s First Attempt at PQ4AI In 2025, the WHO released its first guidance document setting out the standard for an AI tool, specifically, Tuberculosis (TB) computer-aided detection (CAD) solutions^13^. The guidance is plagued by many of the principled concerns discussed elsewhere in this policy brief but also contains unique issues worth highlighting.

First, the guidance falls short by conflating methods commonly used in the context of clinical evidence for (bio)pharmaceutical products with those necessary for external validation of a model. The calculation to derive the recommended number of cases for an effective validation study fails to acknowledge recent advances in the science of model evaluation studies^14,15^. Whilst readily addressable, it does suggest a notable lack of technical understanding of where and how the PQ will need to adapt to accommodate AI, and the fact that the nature of the technology can necessitate a tailored approach to each product.

Second, the guidance, as is standard for PQ requirements, immediately puts discussion of clinical utility (i.e., effectiveness or benefit) out of scope. As noted, this is not a departure from standard practice, but the nuances of TB CAD make this more problematic than usual. The cost-effectiveness of such solutions has been hotly debated^16^, and there is substantial evidence to suggest that they are not^17,18^. It seems odd for an organization that pioneered the concept of ‘best buys’, to have selected a highly contentious solution as its first foray into PQ4AI, especially at a time of extremely constrained resources.

Finally, even if the PQ process were fit for purpose, given the prolific implementation of TB CAD tools around the world, and the numerous products already approved by stringent regulatory authorities^19^, it is unclear what PQ actually adds in the context of TB CAD?

## What is Prequalification (PQ)?

Created to guide UNICEF vaccine procurement in the 1980s and formally expanded to medicines in 2001, later adding diagnostics (2010) and then vector control innovations^2,3^, the WHO Prequalification (PQ) program assures the safety, quality, and efficacy of health innovations. Products that pass the vetting procedure (comprising an assessment of their performance, conformity with international quality standards, and suitability for use in LMICs) are publicly listed, enabling national regulators and international procurers to leverage WHO’s assessment rather than conducting full, independent reviews. In other words, the PQ programme exists to inform multilateral procurement and serve as a reference agency for LMIC regulators operating reliance models. Traditionally, products can be prequalified via two pathways: an abridged process for those already approved by a “WHO-listed authority” (e.g., U.S. FDA, EMA, etc.), or a comprehensive review directly by WHO. While PQ facilitates market access, national authorisation is still required in each country. When carried out in partnership with WHO, this is called the ‘Collaborative Registration Procedure’. Thus, PQ is not intended to replace country-level regulation, but rather to serve as a facilitative tool. The PQ process has been comprehensively reviewed elsewhere^2^.

## What PQ Might Add in the Current AI Regulatory Context

The WHO Prequalification (PQ) programme has consistently demonstrated its ability to respond promptly to emerging global health issues. During the COVID-19 pandemic, it listed 11 vaccines, 42 diagnostics, and prequalified 17 medicines, helping to avert over five million deaths^4^, and demonstrating how the programme can fill a critical gap in the necessary oversight of novel, life-saving products to ensure they reach the people most in need of them. AI-as-a-medical-device presents a similar challenge, with a non-trivial opportunity to improve health and wellbeing, but with an outsized risk if introduced into emerging markets with adequate oversight (both pre- and post-market authorisation). At present, there is only one Sub-Saharan African regulator (Ghana) that is currently systematically reviewing AIaMD products, and no national authority in the region has yet achieved WHO maturity level three (ML3) for medical devices^5^; the threshold that defines a reliable, well-functioning system that can assure safety, quality, and effectiveness across a product’s lifecycle^6^. The relative infancy of the field (of AIaMD regulatory science), combined with the global scarcity of AI regulatory science expertise, means that it will likely take several years to achieve a similar step shift in LMIC oversight capabilities for AIaMD. Thus, the expansion of the PQ programme, if it can leverage the limited pre-existing expertise, is a compelling solution to fill the current gaps.

## The Potential Pitfalls of Prequalifying AI Like It’s a Medicine

The issue is not that the PQ model is a fundamentally bad idea; it has been highly effective for static products, such as medicines and vaccines. However, the current approach faces fundamental challenges when applied to more dynamic AI-based health technologies. Here we describe just one of many.

Current PQ procedures rely heavily on the assumption that a meaningful generalizability guarantee can be derived from clinical validation studies. While this holds (somewhat) true for (bio)pharmaceuticals, it has been repeatedly identified as an issue for AIaMD. In other words, an AI model’s “efficacy” is not a fixed property in the same way a drug’s efficacy is (given the highly conserved biology on which it acts); the former fluctuates with the data fed into it and the conditions under which it’s used, in a much more profound way that the differences we might see due to context-related or pharmacogenetic variation in drug performance. This performance degradation, as real-world data deviates from their training data, is known as drift and limits generalizability^7,8^. In essence, navigating the clinical evidence requirement set out in the PQ technical specification isn’t even a useful litmus test, let alone a true performance guarantee, and runs the risk of providing potentially problematic assurances.

The solution to the aforementioned problem of drift is just as awkward for the PQ programme. To address the issue of drift, regulators such as the FDA have developed innovative solutions, including predetermined change control plans (PCCPs), to enable agreed-upon corrective actions to be taken in response to undesired changes in performance^9^. The underlying paradigm shift is an acknowledgement that an AI tool’s safety and effectiveness are not a one-time evaluation but an ongoing process. The result of utilising a PCCP is that a static PQ listing risks ultimately being at odds with local regulators, given that the local products would eventually differ from the WHO prequalified solution. At minimum, PQ4AI in its current form isn’t fit for purpose; at worst, it acts as a disincentive for local NRAs to adopt harmonised guidance that doesn’t fit the mould of the programme.

## Reimagining PQ4AI

The PQ framework could be adapted to maximize its benefits (global trust and efficiency) while mitigating risks. At a minimum, there is a precedent for several changes that would address some of the issues mentioned above. However, there is also an opportunity for a profound reform of PQ4AI, wherein WHO transitions from acting as a pseudo-regulator to instead leaning into its role as a neutral broker (of quality-assured, highly sensitive information).

### Targeted Reform of PQ4AI

In this model, the WHO would continue to assess manufacturers’ compliance with core international harmonised standards relevant to AIaMD, including ISO 13845, IEC 62304, and ISO 42001, among others, to relieve some of the burden from individual country regulators. Rather, the focus would be on ‘borrowing’ features of the vector control and vaccine PQ programmes, which would provide substantial value if adopted as part of WHO’s current approach to PQ4AI. Firstly, the current “gatekeeping” function of the WHO PQ programme, which requires explicit solicitation of products, can distort the market and introduce unnecessary delays. Following the precedent of the vector control PQ programme^2^, manufacturers should be able to actively engage the WHO team to determine the appropriate pathway for prequalification of an AI-based tool. This would accelerate the submission of novel AI solutions with demonstrated public health value. Secondly, the vaccine PQ programme has a unique requirement wherein the “national regulatory authority (NRA) of the producing country [has] to be functional as a prerequisite for acceptance of submissions from manufacturers from that country”^2^. In the context of AIaMD, we would propose an alternative approach: given the need for local post-market safety monitoring, the NRA of the country in which the product is to be deployed must meet a specific standard for WHO’s prequalification to be a valid predicate (for recognition or reliance). The recently published ‘Global Benchmarking Tool for Medical Devices’ provides a unique platform through which to operationalize this recommendation^10^; specifically, if a dedicated annexe was to be introduced that articulates the minimum requirements of an NRA to rely on a PQ4AI assessment, with detail around what constitutes sufficient post-market safety/surveillance infrastructure in the context of AIaMD deployment. This approach would help address the limitations of one-off evaluation studies, by ensuring that any new issues that arise during real-world use are captured and reported to the regulator. Combined, these two modest changes would make the PQ4AI programme significantly more relevant to the current AI4health ecosystem.

### Overhaul of PQ4AI

Investing in a comprehensive PQ4AI programme (wherein WHO is assessing conformity against AIaMD relevant standards) runs the very real risk of distracting from the critical task of effectively operationalising regional centres of excellence (e.g., the African Medicines Agency and its partner NRAs) to do the same; at a time when global development resources are at historic lows, hard decisions must be made on where to allocate the available financing, and should be based primarily on which actions create the most durable solutions. As such, we believe the better path is a more profound reform of the WHO’s role in the space.

One way in which WHO could carve out a meaningful role for itself is to focus on cutting-edge innovations, which are not going to be addressed by NRAs in emerging markets in the short to medium term (unlike conformity assessment against well-established pre-market submission requirements). Take Generative AI (GenAI)-based product “master files” – confidential dossiers containing the technical underpinnings of the solution, such as the foundation model architecture, training datasets, and documentation of the model development processes – much of which is proprietary. Companies will undoubtedly be hesitant to share this information, but there is a clear precedent from the sharing of such files relating to vaccine adjuvants with regulators globally, that a trusted global body, such as the WHO, could justify a similar procedure for foundation models. The WHO’s role would be to rigorously audit these files, checking that the model was developed in accordance with good machine learning practices^6^, verifying the claimed performance on the provided datasets, and possibly running independent in silico tests (e.g., red teaming to understand model vulnerabilities and risks)^11^. This technical verification by WHO would ensure the algorithm meets certain global standards of quality, analogous to how PQ inspects the quality of manufacturing for drugs. Coupled with the WHO’s work on global digital trust architectures, a novel service function emerges. Just as the Global Digital Health Certification Network is used to enable cross-border verification of health documents (such as COVID-19 vaccination certificates)^12^, a similar concept could underpin the WHO’s new role in facilitating GenAIaMD regulation. For instance, once a GenAI model’s technical dossier is approved and one or more countries report successful local validation, the WHO could issue a digital certificate for that model. This certificate (perhaps linked to the model’s cryptographic hash or version ID) could be recognized across jurisdictions. If another country wants to adopt the GenAI, they could query WHO’s system to confirm they have the certified version, then focus only on bridging studies to ensure it works in their population. This trust network would prevent each country from having to repeat the full due diligence process, while still requiring evidence of local effectiveness. As such, it is akin to the Collaborative Registration Procedure used today for medicines, but updated for the world of GenAI.

## Conclusion

Prequalification for AI-based health technologies should neither be an uncritical transposition of the pharmaceutical model nor an entirely discarded idea. Instead, it should evolve to meet the needs of the technology; a mechanism that necessitates the rigour of local clinical evaluation (and appropriately devolves the oversight of this responsibility to NRAs), whilst providing a depth of technical scrutiny (unachievable by most due to its complexity), delivered through a globally trusted platform. In doing so, WHO can help ensure that as artificial intelligence tools permeate healthcare, they do so safely, effectively, and equitably, with both global assurance and local accountability effectively integrated.

## Data Availability

No datasets were generated or analysed during the current study.
